# A methodology to implement a closed-loop feedback-feedforward level control in a laboratory-scale flotation bank using peristaltic pumps

**DOI:** 10.1016/j.mex.2023.102081

**Published:** 2023-02-16

**Authors:** Paulina Quintanilla, Daniel Navia, Felipe Moreno, Stephen J. Neethling, Pablo R. Brito-Parada

**Affiliations:** a Department of Earth Science and Engineering, Royal School of Mines, Imperial College London, South Kensington Campus, London SW7 2AZ, United Kingdom; b Departamento de Ingeniería Química, Universidad Técnica Federico Santa María, Campus San Joaquín, Santiago, Chile

**Keywords:** Closed-loop level control, Feedback-feedforward control, Froth flotation, Flotation control, Integrating process, Laboratory-scale flotation bank, SIMC tuning, Level control of tanks in series using peristaltic pumps

## Abstract

This paper describes the implementation of a level control strategy in a laboratory-scale flotation system. The laboratory-scale system consists of a bank of three flotation tanks connected in series, which mimics a flotation system found in mineral processing plants. Besides the classical feedback control strategy, we have also included a feedforward strategy to better account for process disturbances. Results revealed that the level control performance significantly improves when a feedforward strategy is considered.

This methodology uses peristaltic pumps for level control, which has not been extensively documented even though: (1) peristaltic pumps are commonly used in laboratory-scale systems, and (2) the control implementation is not as straightforward as those control strategies that use valves. Therefore, we believe that this paper, which describes a proven methodology that has been validated in an experimental system, can be a useful reference for many researchers in the field.

• Preparation of reagents to ensure that the froth stability of the froth layer is representative of an industrial flotation froth.
• Calibration of instruments – convert the electrical signal from PLCs to engineering units.
• Tuning PI parameters using SIMC rules by performing step-changes in each flotation cell.

## Graphical abstract

**Figure:**
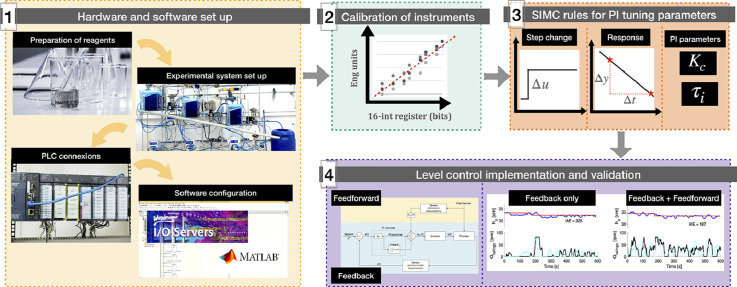

Specifications table

**Table:** 

Subject area:	*Engineering*
More specific subject area:	Process control
Name of your method:	Level control of tanks in series using peristaltic pumps
Name and reference of original method:	Not applicable
Resource availability:	Software: Proficy Machine Edition, GEHCS I/O Server, MATLAB

## Background on level control for the flotation process

Level control plays an important role in many processes in the chemical industry. Particularly for froth flotation, the largest tonnage mineral separation process, the importance of level control relies upon the fact that it affects the purity of the separation product (i.e. the concentrate grade), as well as the recovery of the valuable mineral particles [1,2]. The regulatory level control can be performed by manipulating either the position of a valve's plug or pump speed to change the tailings flowrate of a cell. While much research has focused on level control using valves (e.g. [5], [6], [7]), work reported for level control via pump speed is scarce (e.g. [8]). Moreover, unlike the present work, most studies only present theoretical aspects of the control applied in simulation, without a practical application in an experimental system. For a deeper discussion on the methodologies for level control in flotation cells, the reader is referred to [3,4].

The use of pumps for level control is also known as averaging control. It is considered an integrating process due to its inability to achieve a steady state without a controller's intervention; unlike self-regulating processes, the integrating process will reach a steady state only using a closed-loop feedback controller design. A Proportional-Integral-Derivative (PID) control strategy is the most extended feedback technique for regulatory control in industrial processes. One of the most common mathematical expressions for a PID controller is:

(1) $$u\left(t\right)=K_{c}\left(e\left(t\right)+\frac{1}{\tau_{i}}\int_{0}^{t}e\left(\tau \right)d\tau +\tau_{d}\frac{de\left(t\right)}{dt}\right).$$

Eq. (1) is also known as serial or standard structure, where u(t) is the control signal for the manipulated variable, e(t) is the error defined as the difference between the desired setpoint ($y^{sp})$ and the actual value of the variable (y(t)). $K_{c}$, $\tau_{i}$ and $\tau_{d}$ are tuning parameters, corresponding to the controller gain, integral time, and derivative time, respectively. It should be noted that in some cases a proportional-only (P) or proportional-integral (PI) controller is sufficient to obtain a reasonably good level control. For example, in the case of froth flotation, PI controllers are generally used to maintain pulp level at the desired setpoint [6,[9], [10], [11].

Much attention should be paid to the tuning of the controller parameters ($K_{c},\tau_{i}$ and $\tau_{d}$); otherwise, the closed-loop response may become unstable. Extremely slow or too-aggressive controllers should be avoided. One way to properly tune the parameters for an integrating process, like the level control via pumps, is by using the Simple Internal Model Control (SIMC) rules [12]. The integral term rule in the SIMC method is modified to improve the disturbance rejection for integrating processes.

This paper describes the design and experimental implementation methodology of a closed-loop feedback-feedforward strategy for level control in an integrating process. The integrating process consists of a three-cell laboratory-scale flotation bank. The tuning parameters were calibrated using the SIMC rules described in [12]. The inclusion of the feedforward strategy in the traditional feedback control strategy was assessed by evaluating the total sum squared error (SSE) in each flotation cell. It should be mentioned that the final aim of this work is to ensure a robust regulatory control layer to implement a superior optimal control layer based on advanced controllers, such as an economic model predictive control strategy. Such an advanced control strategy will need a flotation dynamic model, as found in [13,14].

## Materials

### Experimental rig

**Fig. 1:**
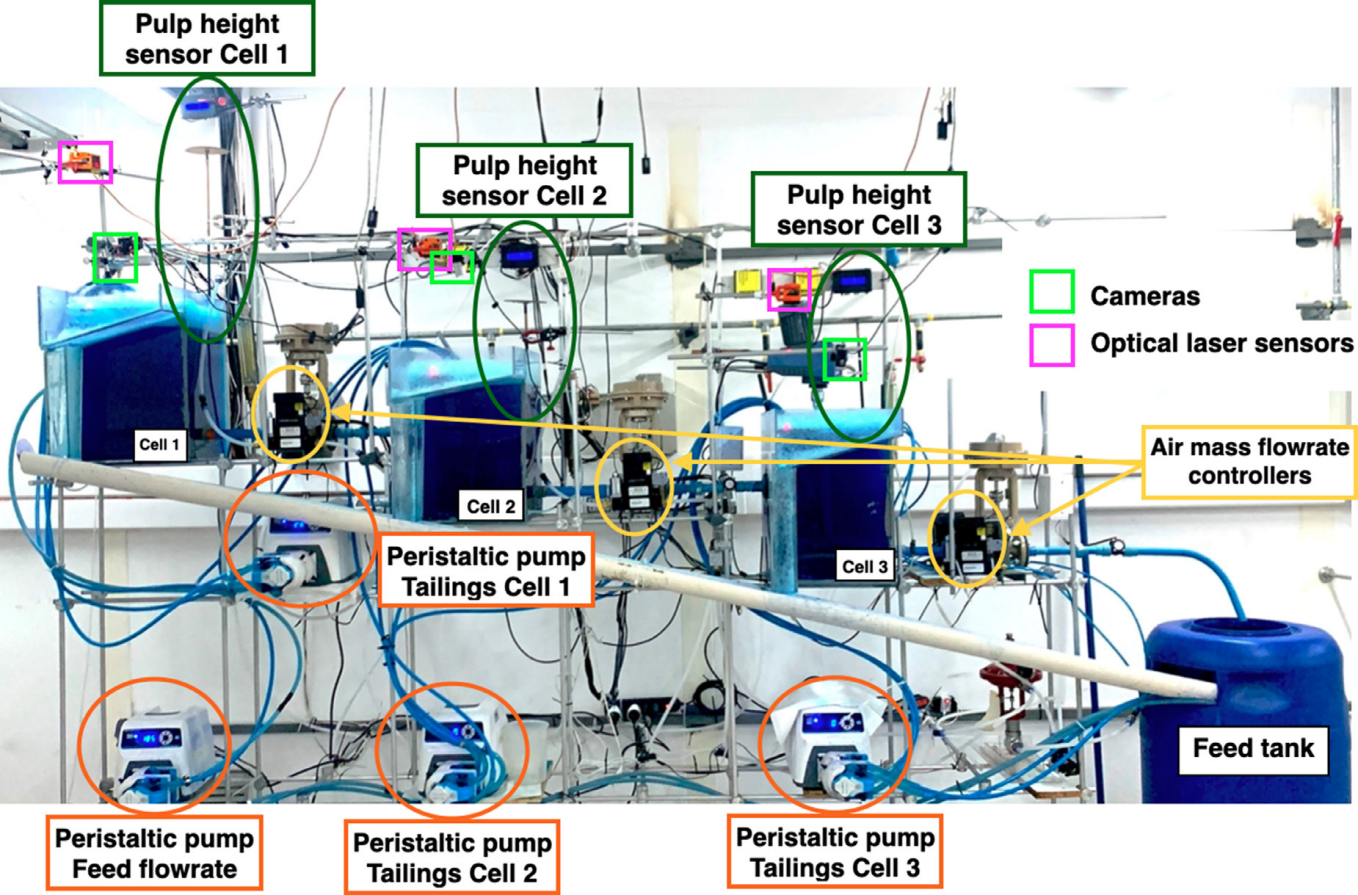
Experimental flotation bank with three cells in series. Peristaltic pumps, level sensors, air mass flowrate controllers, optical level sensors and cameras are highlighted. The optical level sensors and cameras are used to measure froth stability through air recovery, as explained in the “Preparation of reagents” subsection.

**Fig. 2:**
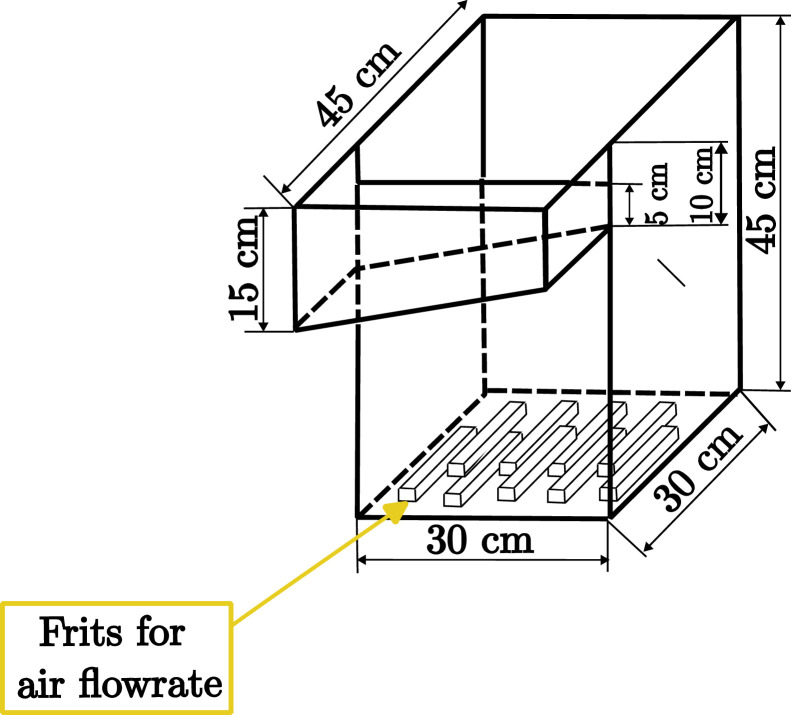
Cell dimensions. Frits are located at the bottom of the cells to allow for small bubbles to be formed when air passes through them.

The experimental rig consists of a three-cell flotation bank interconnected in a closed loop, as shown in Fig. 1. The cells are interconnected such that the tailings flowrates of each cell are the feed to the next one. The first cell is fed from the feed tank, which receives the tailings from the third cell, so that flow is recycled. A compressed air injection system is located at the base of each cell, which passes air through frits (mesh hole size of 20 μm) to form small bubbles. The cells are rectangular cuboids made of acrylic. The cross-sectional area of each cell is 300 × 300 mm and the height is 450 mm, as shown schematically in Fig. 2.

**Fig. 3:**
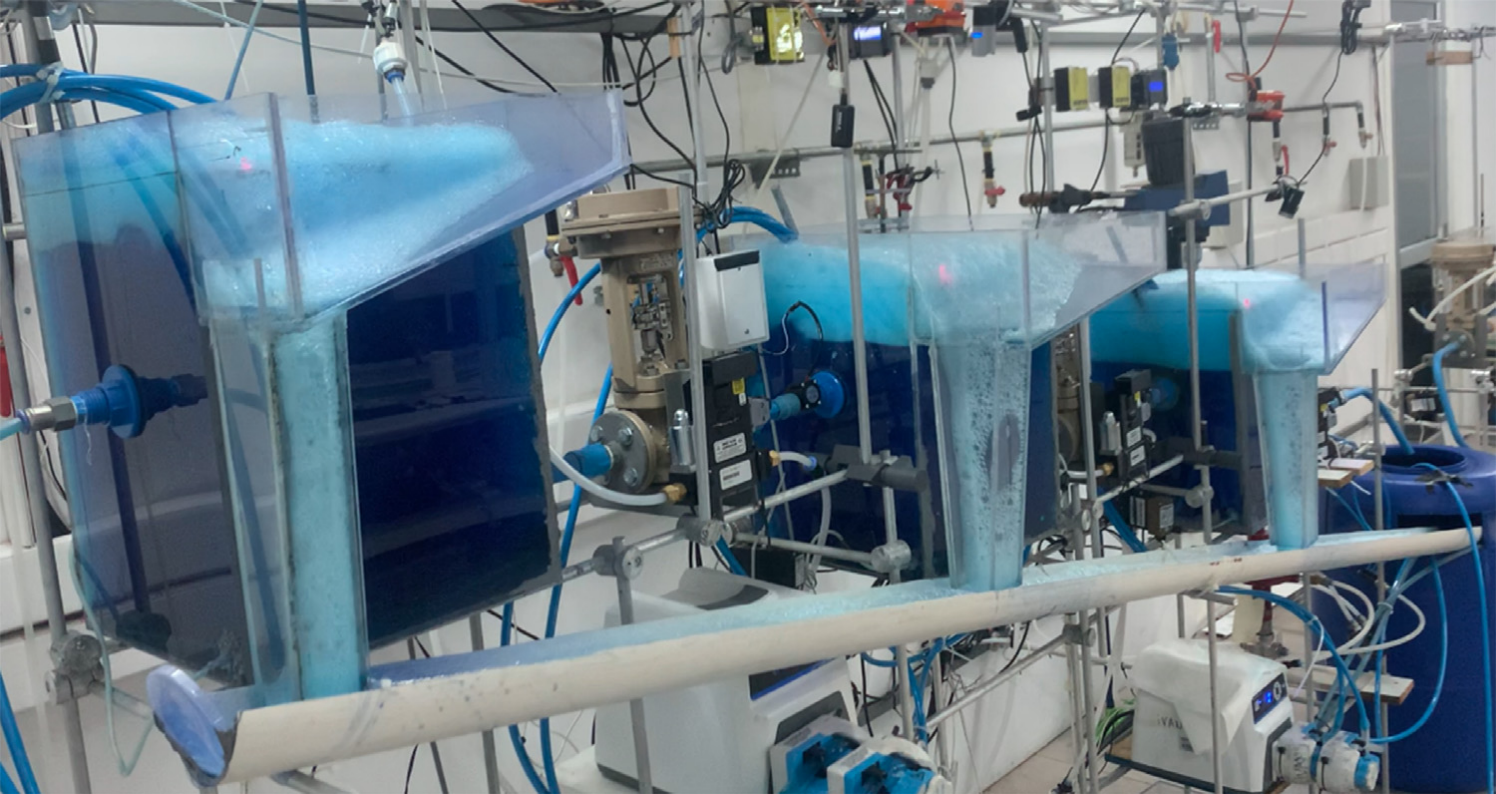
Experimental system with froth overflowing from the top of each bank.

Fig. 3 shows the overflowing froth is collected in a cross-cut pipe to the feed tank. The continuous recycling of the concentrate flowrates in the feed tank allows for steady-state conditions to be reached. The feed tank capacity is 130 litres.

#### Control instrumentation

**Fig. 4:**
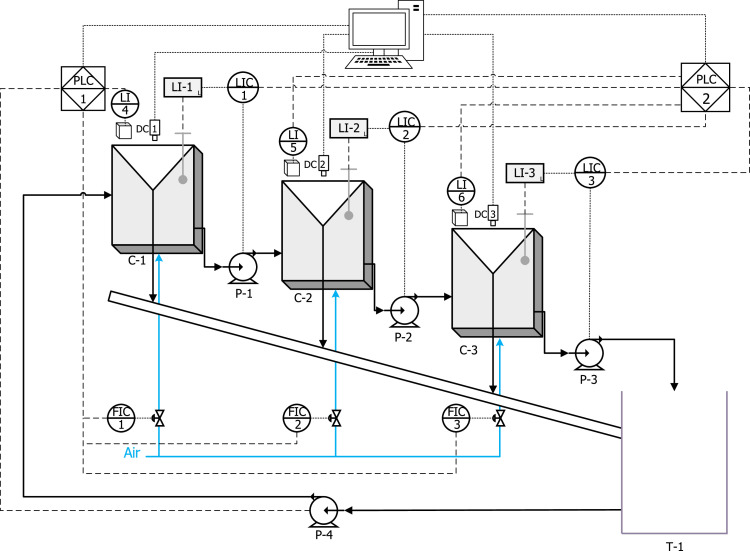
P&ID of the experimental rig for the control level. P-1/4 are peristaltic pumps, C-1/2/3 are the flotation cells, LI-1/2/3 are the level sensors (floating system + optical level sensors), FIC-1/2/3 are the air massflow controller, and T-1 is the feed tank. All instruments are connected to General Electrics PLCs, which are connected to a stationary computer. LI-4/5/6 are optical laser sensors to measure the froth heights over the cell lips, and DC-1/2/3 are digital cameras. Optical lasers and cameras are used to measure froth stability through air recovery, as explained in the “Preparation of reagents” subsection.

Fig. 4 shows a piping and instrumentation diagram (P&ID) of the experimental rig. The first cell, C-1, is fed with a mixture of water and reagents driven by the peristaltic pump, P-4, from the tank, T-1. The tailings flowrate from C-1 feeds the second cell, C-2 via a peristaltic pump, P-1; and the tailings from C-2 go to the third cell, C-3, via a peristaltic pump, P-2. The tailings flowrate of C-3 is discharged into tank T-1, via a peristaltic pump, P3. The concentrate flowrates from the three cells are combined and discharged into T-1. The air mass flowrate controllers are represented as FIC-1/2/3. The level control was conducted through the peristaltic pumps with double heads to ensure adequate residence time in each cell. Pulp levels were measured using a floating system connected to optical sensors (LI-1/2/3 in Fig. 4) in each cell.

All instruments were connected to General Electric Programmable Logic Controllers (PLCs), models ALG223 and ALG392. The ALG223 model was used to ‘read’ signal data sent from sensors in the system. These signals from the sensors are also known as analogue inputs. The ALG392 model was used to send data from software, through the PLC, to an actuator; these are also known as analogue outputs. The analogue inputs in this study correspond to the level and air flowrate sensors, while the analogue outputs are the revolutions per minute (rpm) of the peristaltic pumps, as well as the control valve of the air mass flowrate controllers. Both analogue inputs and outputs were configured to 4–20 mA current loop signals. A summary of the instruments is presented in Table 1, including their brands/models.

**Table 1:** Control instrumentation.

Instrument	Brand	Type	Tag in P&ID
Peristaltic pumps	Masterflex L/S 77,916–10. Range: 0–600 rpm	Actuators	P-1/2/3/4
Air mass flowrate controllers	Aalborg Compact Gas Mass Flow Controller. Range 0–100 LPM	Actuators and sensors	FIC-1/2/3
Optical level sensors	N/A	Sensors	LI-1/2/3
Programmable logic controllers (PLCs)	GE Fanuc. Models: IC693ALG392 (controllers and actuators) and IC693ALG223 (sensors)	PLC	PLC-1/2

#### Control software

The control logic was programmed in ladder language using General Electrics Proficy Machine Edition 7.0 software. The control variables were linked in Proficy Machine Edition to the corresponding storage spaces in the PLCs. Then, General Electrics I/O Server, called GE Fanuc Host Communications (GEHCS I/O Server), was used to establish communication between Proficy Machine Edition and MATLAB. This communication is particularly useful to calibrate the instrumentation, as well as to implement an online control.

The steps to set up the control instruments in Proficy Machine Edition 7.0 and to build the logic programme are as follows:

1. Once the communication between PLCs and Proficy Machine Edition is established, the modules of the PLC should appear in the left window as shown in Fig. 5:

**Fig. 5:**
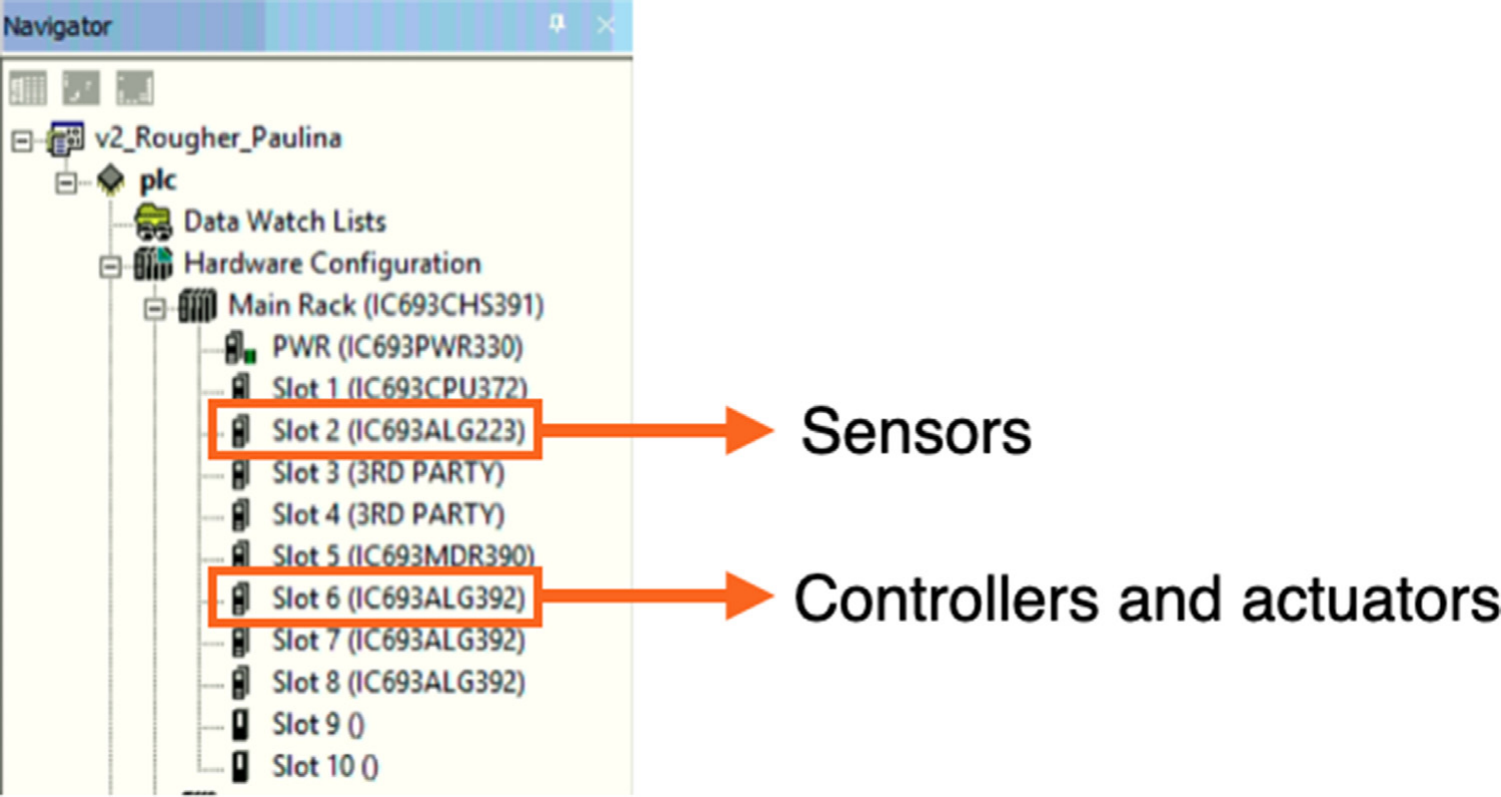
PLC modules in the Proficy Machine Edition 7.0 software.
2. Identify the physical channels (also called ‘terminals’) in the PLCs where each instrument is connected.
3. Assign a ‘Reference Address’ to each physical channel. The Reference Address is the PLC memory location that contains the variable's value, and it depends on the data type. For example, the reference address for sensors is “AI00X”, while for controllers and actuators, the reference address is “AQ00X” (where X is an integer number). Each reference address must be unique for each instrument.
4. Verify that the “input/output channel data” option in Proficy Machine Edition is correctly set up for each reference address (AI00X or AQ00X), according to the instrument requirements. The options for the PLC modules used in this study (IC693ALG223 and IC693ALG392) are 4–20 mA, 0–10 V, −10–10 V, and 0–20 mA.
5. Create a new control logic, using a program block which should appear in the left window, as shown in Fig. 6. This allows for a ladder control strategy to be built. To this end, it is necessary to link each reference address to a block. In this study, we used blocks called MOVE_INT, whose function is to receive power flow and copy data as individual bits from one channel in PLC memory to another [15].

**Fig. 6:**
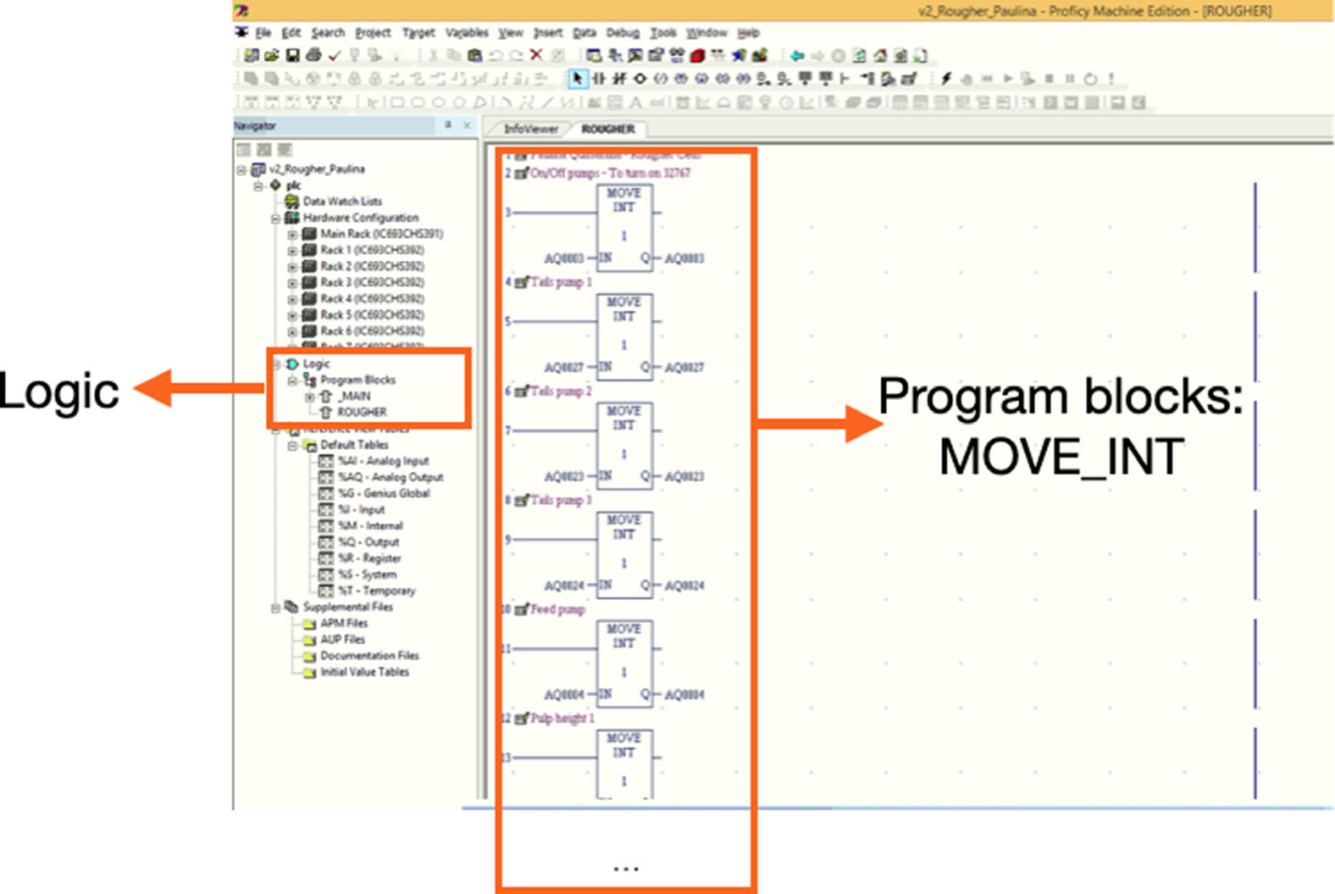
Control logic window. Each reference address must be linked to a MOVE_INT block.

### Preparation of reagents

The experiments were carried out in a two-phase system (water and air) with chemical reagents.

The chemical reagents allowed for a froth layer with sufficient stability to be generated. The feed tank was filled with tap water (from Santiago, Chile), and then the chemical reagents were added in the following order:

(1) Methylene blue at a concentration of 10 ppm,
(2) Methyl isobutyl carbinol (MIBC) at a concentration of 330 ppm,
(3) Frother Flottec F160–10 at a concentration of 20 ppm, and
(4) Xanthan gum at a concentration of 600 ppm.

Methylene blue was used to measure air recovery. Air recovery is a measure of froth stability, and it is defined as the fraction of air entering a flotation cell that overflows the cell lip as unburst bubbles [16,17]. To be able to measure air recovery, optical lasers and cameras were placed at the top of each cell, as described in [14]. The optical lasers are used to measure froth height over the cell lip, and the cameras are used to measure the horizontal overflowing froth velocity using image analysis software. The cameras used in these experiments were digital video cameras (commercially available webcams) that were directly connected to the same computer in which the image analysis software was installed. The optical lasers can measure only opaque surfaces, and since this system was two-phase only (i.e. without solids), methylene blue was needed to produce sufficient opaquest between bubbles at the froth surface. Frother and surfactants, such as Flottec 160–10 and MIBC, are commonly added in flotation systems to control bubble size in the pulp phase, as well as to stabilise the froth phase. In addition, a viscosity modifier, such as xanthan gum, is usually used for laboratory-scale experiments without solids. In this study, commercially available xanthan gum was used for all experiments.

The first three reagents (methylene blue, MIBC and frother) were added directly to the feed tank, which was stirred for 5 min to homogenise the mixture. Due to the high thickening property of xanthan gum, it is recommended to prepare the 600-ppm solution in a smaller container (i.e. external to the system), as it tends to form lumps in the beginning. Once the xanthan gum solution is homogeneous, it must be poured into the feed tank and stirred with an external agitator for an additional 5 min. It should be noted that the water used to prepare the xanthan gum solution was also considered in the final mixture. For example, if the feed tank had a capacity of 100 litres, then the tank should initially be filled with 98 L of water (to which the Flottec-160–10 and MIBC were added), and the remaining 2 L of water should be used for the preparation of the xanthan gum solution. Concentrations in ppm of all reagents should be thus based on a total of 100 litres.

## Procedures

### Calibration of instruments

**Fig. 7:**
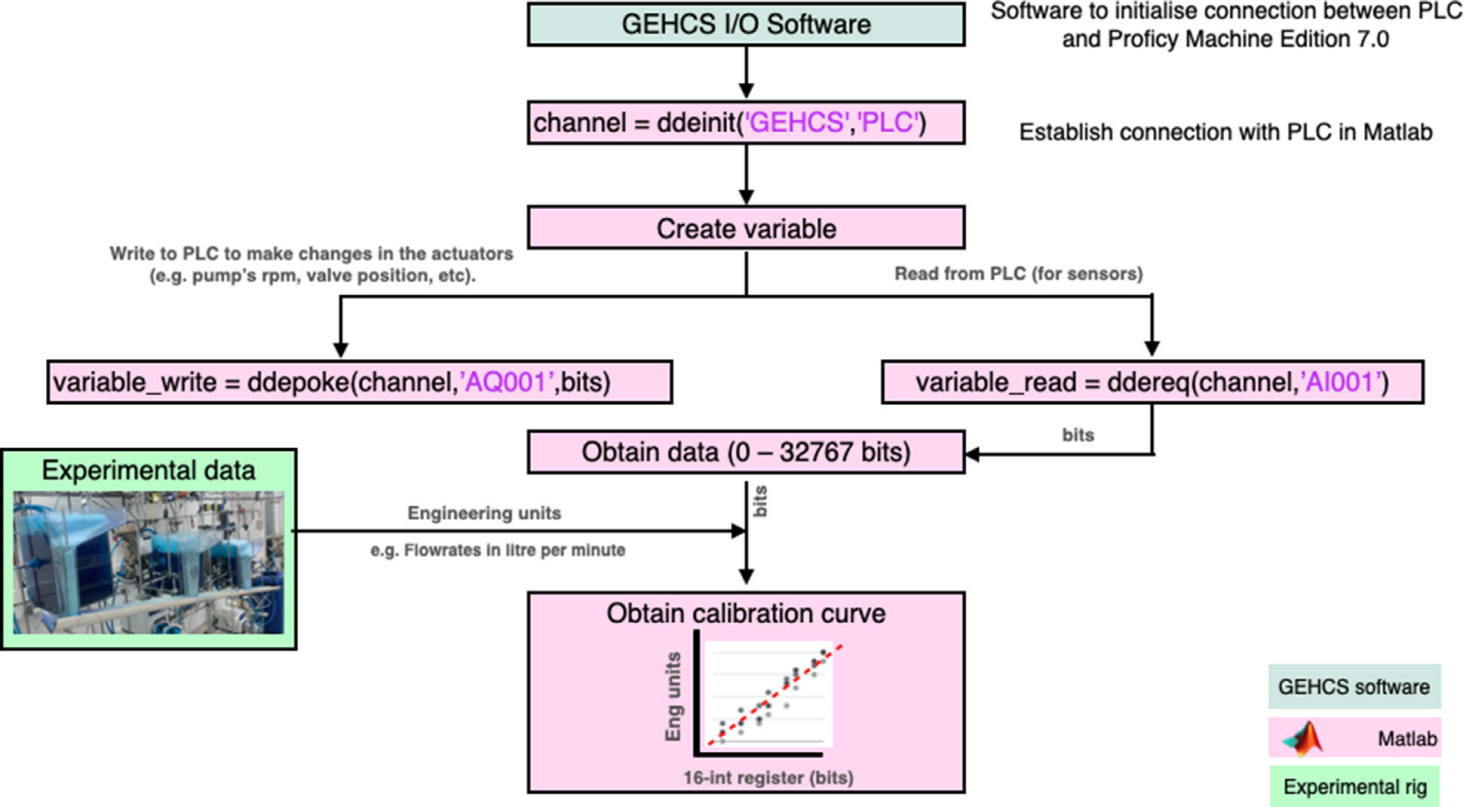
Flowchart for the calibration of instruments. The green box corresponds to the GEHCS I/O Server software, the pink blocks correspond to the procedures made in MATLAB, and the yellow box is the experimental rig.

Once the logic control is set up, the calibration of instruments is performed following the steps shown in Fig. 7. First, communication between GEHCS I/O Server and MATLAB was established using the function *ddeint*. Two additional MATLAB functions were used: *ddereq* and *ddepoke. ddereq* allows ‘reading’ the signals (in 16-bit integer) that come to the PLCs from the sensors. *ddepoke* allows ‘writing’ signals (in 16-bit integer) to the PLCs, which the PLCs then send to the actuators.

These functions allow for the instruments to be calibrated using MATLAB, converting the analogue signals (16-bit integer limit) to engineering units (e.g. the range of level sensors was within 0–39 cm). The 16-bit integer limit uses 16-bit memory data locations, whose valid range for an INT data type (as the ones used in this study) is 0 to 32,767. Each calibration was performed in triplicates to ensure repeatability and for further statistical analysis.

#### Air mass flowrate controllers

The air mass flowrate controllers include a built-in valve and have their own PID control integrated into the system. The air passes into a straight-tube sensor, ensuring accurate and repeatable results. The set points and the output data can be established by either a 4–20 mA signal or 0–5 VDC signal, having an analogue-to-RS converter which allows data collection in the computer. These controllers were set as 4–20 mA signal and had to be calibrated in both ways: *read* from the sensor to convert bits to litres per minute (lpm), as well as *write* from the PLC (in bits) to the built-in valve.

**Fig. 8:**
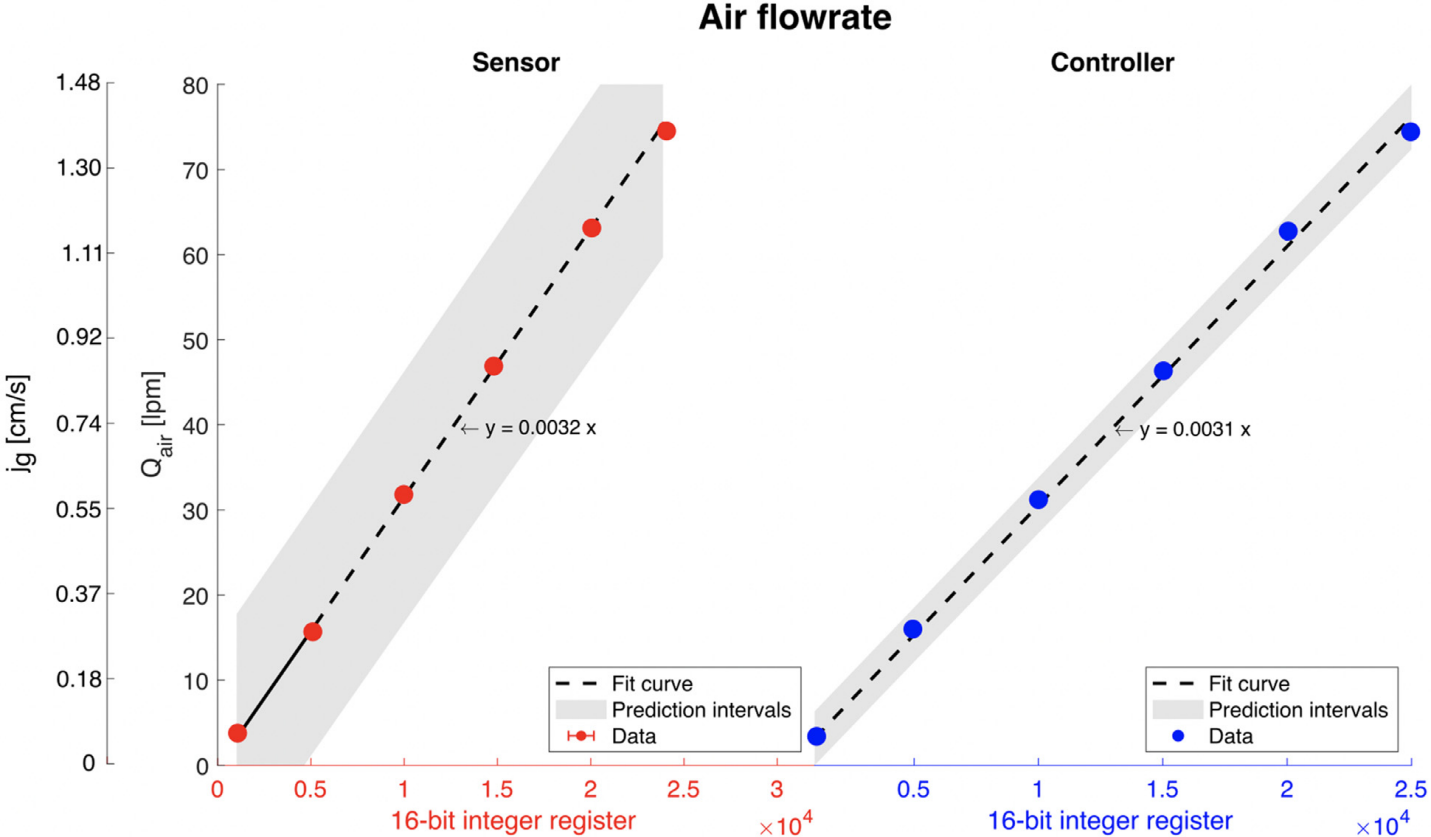
Calibration curves air flowrate controller. The red dots are the experimental data points with their respective error bars corresponding to ± 1 standard deviation in the bits read. The dashed lines are the fit curve (intercept fixed in 0), and the grey-shaded envelopes are the prediction intervals with a 95% confidence level. The blue dots are the flowrates read from the LCD in the sensor.

It was found that the valve did not present hysteresis, having a linear relationship between the bits and the lpm, as shown in Fig. 8. Note that the air controllers are identical for all three cells and therefore the calibration curve shown in Fig. 8 for cell 1 is the same for cells 2 and 3. Additionally, note that in froth flotation, the volumetric air flowrate, $Q_{air}$ (in this case in lpm), is usually divided by the cross-sectional cell area, $A_{cell},$ and it is known as the superficial air velocity,$j_{g}$, which is calculated as $j_{g}=Q_{air}/A_{cell}$.

#### Peristaltic pumps

The calibration procedures of the peristaltic pumps were made for each pump independently. To measure the flowrate coming from the feed tank or tailings, it is necessary to count with a 2-litre graduated cylinder and a chronometer.

The calibration of the pumps must be carried out by two people, following these steps:

1. Ensure that the flotation cell is full of the mixture of water and reagents.
2. One person must be working on the experimental rig and the other on the PC. The person on the experimental rig must hold the graduated cylinder to collect the pump discharge.
3. The person on the PC sends different bits from the Proficy Machine Edition, or MATLAB, is reached (10 s in this case). In this study, the 16-bit integer register (bits) sent to each peristaltic pump were: 5000, 10,000, 20,000, 25,000, and 28,000 bits.
4. Register the volume obtained in 10 s. Then calculate the flowrate as volume/time.
5. Repeat steps 2–4 two more times for each value of bits sent to the pump to obtain triplicates for each operating point.

**Fig. 9:**
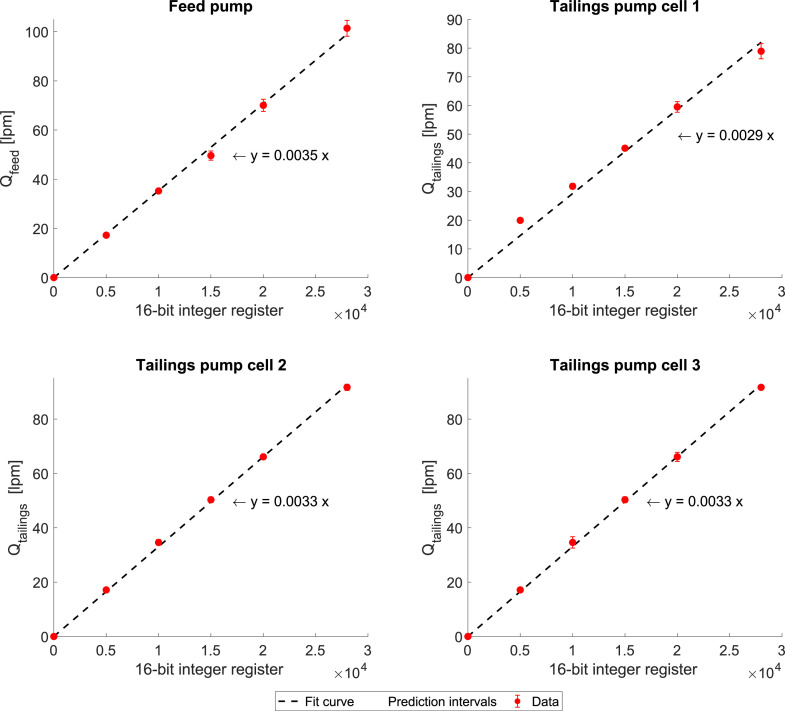
Calibration curves for feed and tailings flowrate pumps. The red dots are the experimental data points with their respective error bars corresponding to ± 1 standard deviation. The dashed lines are the fit curve (intercept fixed in 0), and the grey-shaded envelopes are the prediction intervals with a 95% confidence level.

Fig. 9 shows the calibration curves for the four pumps in the experimental rig. As can be seen, the variability of flowrates in the feed is slightly higher than for the tailings flowrates. This is because it was more difficult to allocate the graduated cylinder to collect the discharge flowrate. However, the variability is marginally minor, and the data allowed for a calibration curve to be built. In all cases, there is a linear relationship between the flowrates and the bits.

#### Level sensors

The calibration procedures for level sensors were carried out by fixing the air flowrate at 47 lpm (i.e. superficial gas velocity $j_{g}$ = 0.87 cm/s) since the level sensor signals are noisier due to the turbulence generated by the air injection. Each sensor was calibrated independently. The steps to calibrate the level sensors are as follows:

1. Fill the flotation cell until the level reaches its maximum.
2. Register the bits sent from the sensor to the PLC and then to the PC (via Proficy Machine Edition or MATLAB).
3. Decrease the pulp level by X cm using the peristaltic pump. In our case, *X* = 2 cm in most cases (see Fig. 10).

**Fig. 10:**
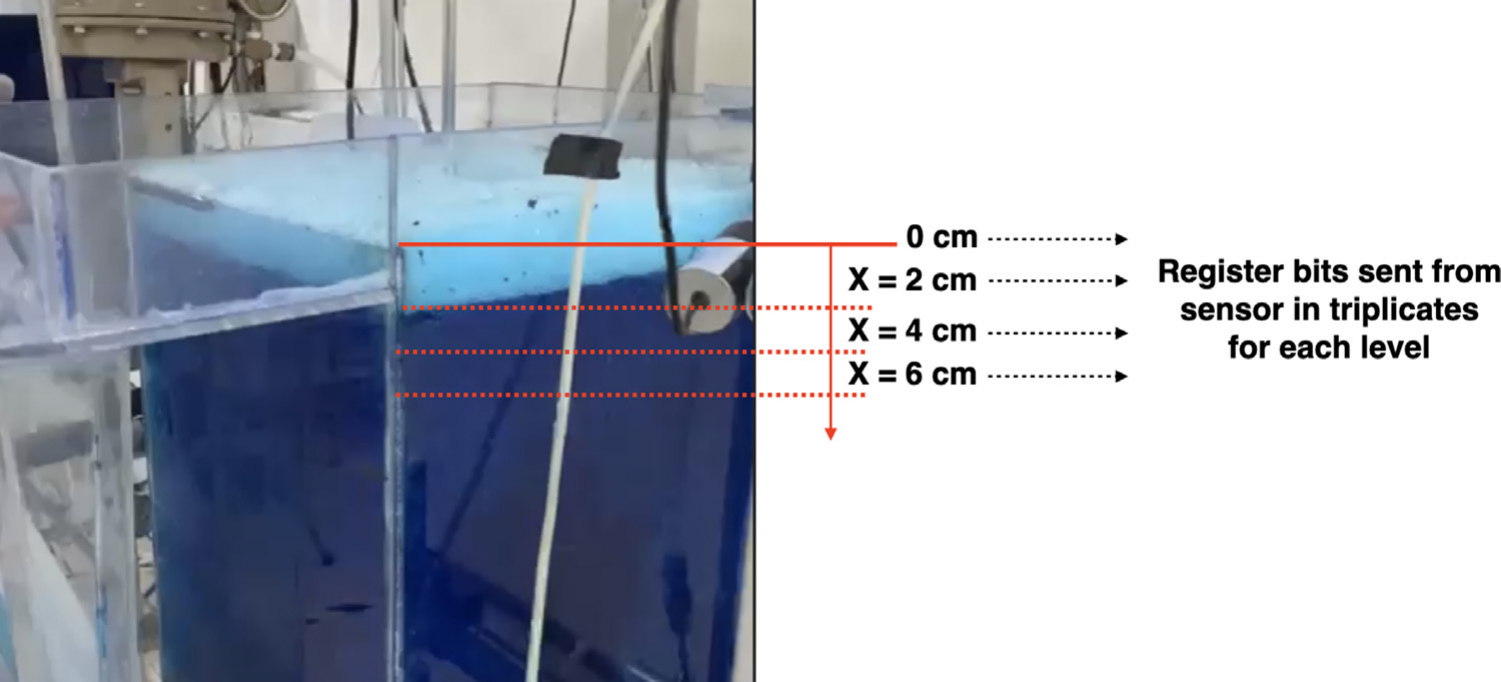
Experimental procedure for calibration of level sensors. Measure different levels by decreasing the pulp level every two cm (or any convenient difference), and register the bits sent from the sensor to the PLC. The scale in this figure does not necessarily represent the actual values.
4. For further statistical analysis, repeat steps 2 and 3 for each level X, to obtain triplicates.

**Fig. 11:**
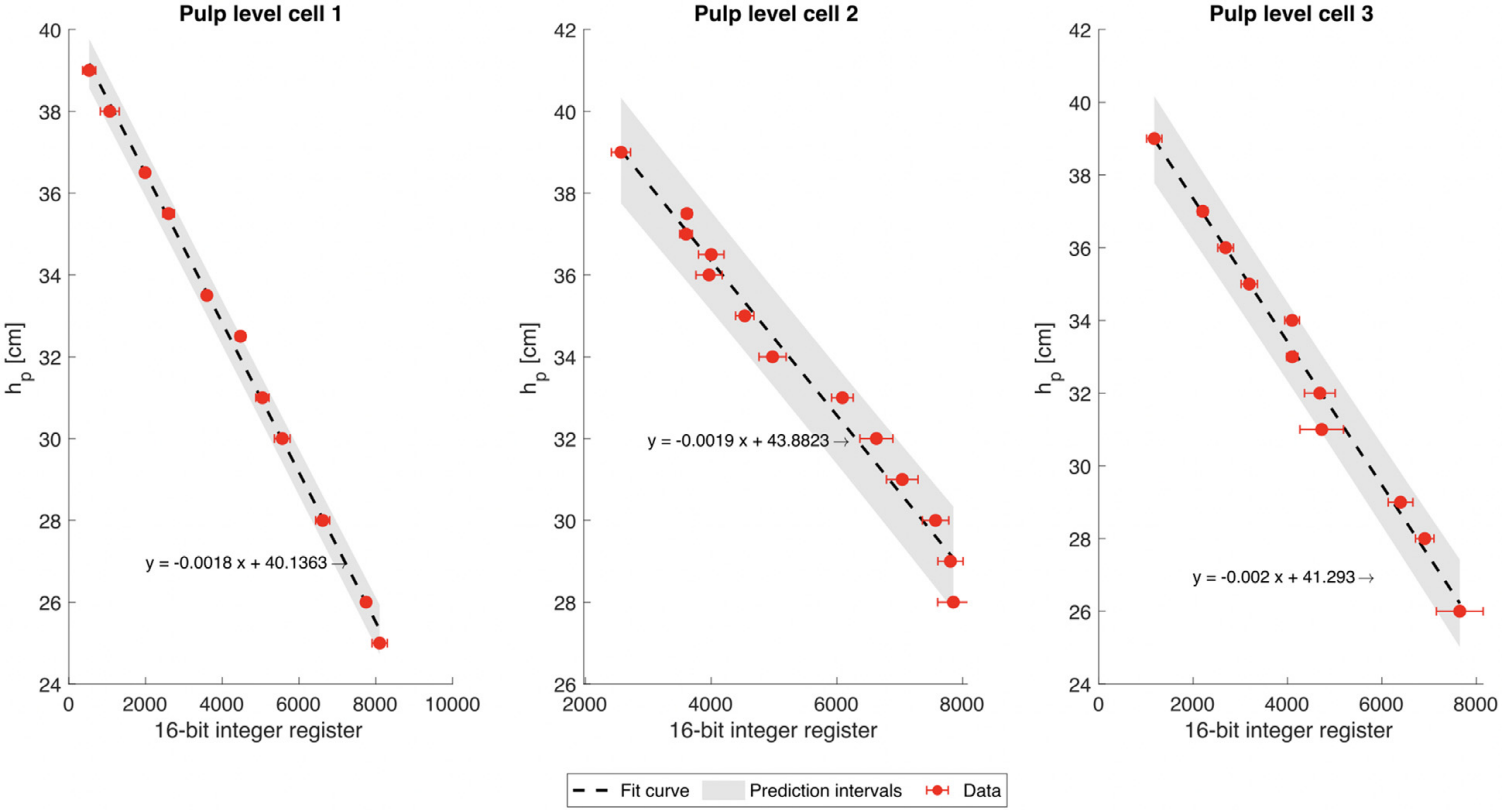
Calibration curves the pulp level sensors ($h_{p}$). The red dots are the experimental data points. The error bars correspond to ± 1 standard deviation of the recorded 16-bit integer register for each fixed-level value. The dashed lines are the fit curve (intercept fixed in 0), and the grey-shaded envelopes are the prediction intervals with a 95% confidence level.

Fig. 11 shows the calibration curves for each level sensor, with their respective variability of the bits that were sent from the level sensors.

### PI parameters tuning using simple internal model control (SIMC) rules

#### Background information

The Simple Internal Model Control tuning rules for a PI controller are based on the following equations [12]:

(2) $$K_{c}=\frac{1}{k}\frac{\tau_{1}}{\tau_{c}+\theta }=\frac{1}{k^{\prime }}\frac{1}{\tau_{c}+\theta },$$

(3) $$\tau_{i}=\min\left\{\tau_{1},4\left(\tau_{c}+\theta \right)\right\}.$$

**Fig. 12:**
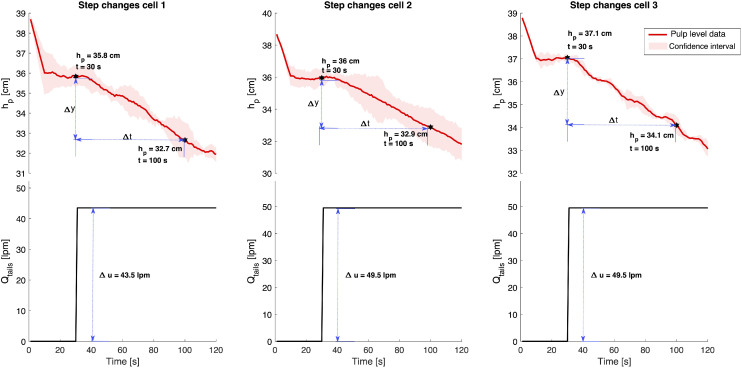
Step changes and pulp level responses ($h_{p}$) to calculate the tuning parameters using the SIMC method. The red lines are the pulp level data, and the black stars are the two points selected to calculate Δy and Δt.

The parameters $k^{\prime }$and θ were obtained from open-loop step-response graphs of each cell individually, shown in Fig. 12. $k^{\prime }$ is the slope of the integrating response, and θ is the delay time where the output (level) does not change after applying a step change in the input. $\tau_{c}$ is the only tuning parameter. The corresponding first-order transfer function for an integrating process ($G_{p}\left(s\right)$), that an input, U(s), to an output, Y(s), with time delay (θ) is:

(4) $$G_{p}\left(s\right)=\frac{Y\left(s\right)}{U\left(s\right)}=\frac{k}{\left(\tau_{1}s+1\right)}e^{-\theta s}$$

where k is the process gain and $\tau_{1}$ is the lag time constant. The derivation of Eq. (4) can be found in the “Additional information” section at the end of this paper. It is important to note that only a Proportional-Integral (PI) controller was used for each cell i, for i=1,2,3 (i.e. $\tau_{d}=0$ in Eq. (1)) due to the difficulty of tuning the controller parameters for level control. The derivative time, $\tau_{d}$, is recommended to be different from zero for level control with a dominant second-order behaviour (not this case) [18].

#### Experimental procedure for parameter tuning

The experimental procedure is straightforward. The step changes for the peristaltic pumps were performed in triplicates to obtain good representativity of level changes. Fig. 12 shows the step changes and the response in level for each cell. As can be seen, two points in level response must be chosen. These two points will be used to calculate Δy and Δt, which are used to calculate $k^{\prime }$ of Eq. (2) as follows:

(5) $$k^{\prime }=\frac{\Delta y}{\Delta t\cdot \Delta u}$$

Then, the response time is used to calculate θ in Eq. (2). This was also calculated graphically and corresponds to the time when the output (level) does not change. In this case, *θ* = 7,6 and 3 s for cells 1, 2 and 3, respectively. Given an initial assumption that $\theta =\tau_{c}$, $K_{c}$ was calculated using Eq. (2) for each cell independently. For robustness, it must be ensured that $\tau_{c}\geq \theta$. Additionally, for an integrating process like this one, it is assumed that $\tau_{i}=\tau_{1}$ (Eq. (3)), and that:

(6) $$\tau_{i}\geq 4\left(\tau_{c}+\theta \right)=8\theta$$

The PI control parameters were calculated from Eqs. (2) and 3, assuming that $\tau_{c}$ was equal to θ, and that $\tau_{1}=8\theta$ (Eq. (6)) [18,19]. Then, $\tau_{c}$ was iterated until reasonably good regulatory control is achieved. It should be noted that a too large $\tau_{i}$ results in poor disturbance rejection, but slow oscillations can be obtained when it is too small. Thus, it is required to ensure that $\tau_{i}\geq 4\left(\tau_{c}+\theta \right)$.

It must be emphasised that the parameters $K_{c}$ and $\tau_{i}$ obtained from Eqs. (2) and 3 were used as initial points. To obtain the definite tuning parameters, trial-and-error experiments were performed, implementing a feedback-feedforward strategy (as shown in the next section). The final tuning parameters and the corresponding first-order model of each cell are given in Table 2.

**Table 2:** Tuning parameters and transfer function of each flotation cell.

Parameter	Units	Cell 1	Cell 2	Cell 3
$k^{\prime }$	$\text{cm}s^{-1}\text{lp}m^{-1}$	−0.001018	−0.001018	−0.001018
*θ*	s	7	6	3
$\tau_{c}$	s	7	6	8
$\text{Initial}\;K_{c}^{\left(1\right)}$	$\text{lpm}\;\text{c}m^{-1}$	−70.2	−81.8	−78.1
$\text{Final}\;K_{c}^{\left(1\right)}$	$\text{lpm}\;\text{c}m^{-1}$	−62	−76	−85
$\text{Initial}\;\tau_{i}^{\left(1\right)}$	s	56	48	52
$\text{Final}\;\tau_{i}^{\left(1\right)}$	s	56	30	44
$G_{p}\left(s\right)$	–	$\frac{-0.001018e^{-7s}}{s}$	$\frac{-0.001018e^{-6s}}{s}$	$\frac{-0.001018e^{-3s}}{s}$

(1) Note that “Initial $K_{c}$” and “Initial $\tau_{i}$” refers to the value calculated from Eq. (2) and 3, respectively; while “Final $K_{c}$” is the actual value obtained after several trial-and-error experiments.

### Feedback-feedforward PI control implementation using SIMC tuning rules

**Fig. 13:**
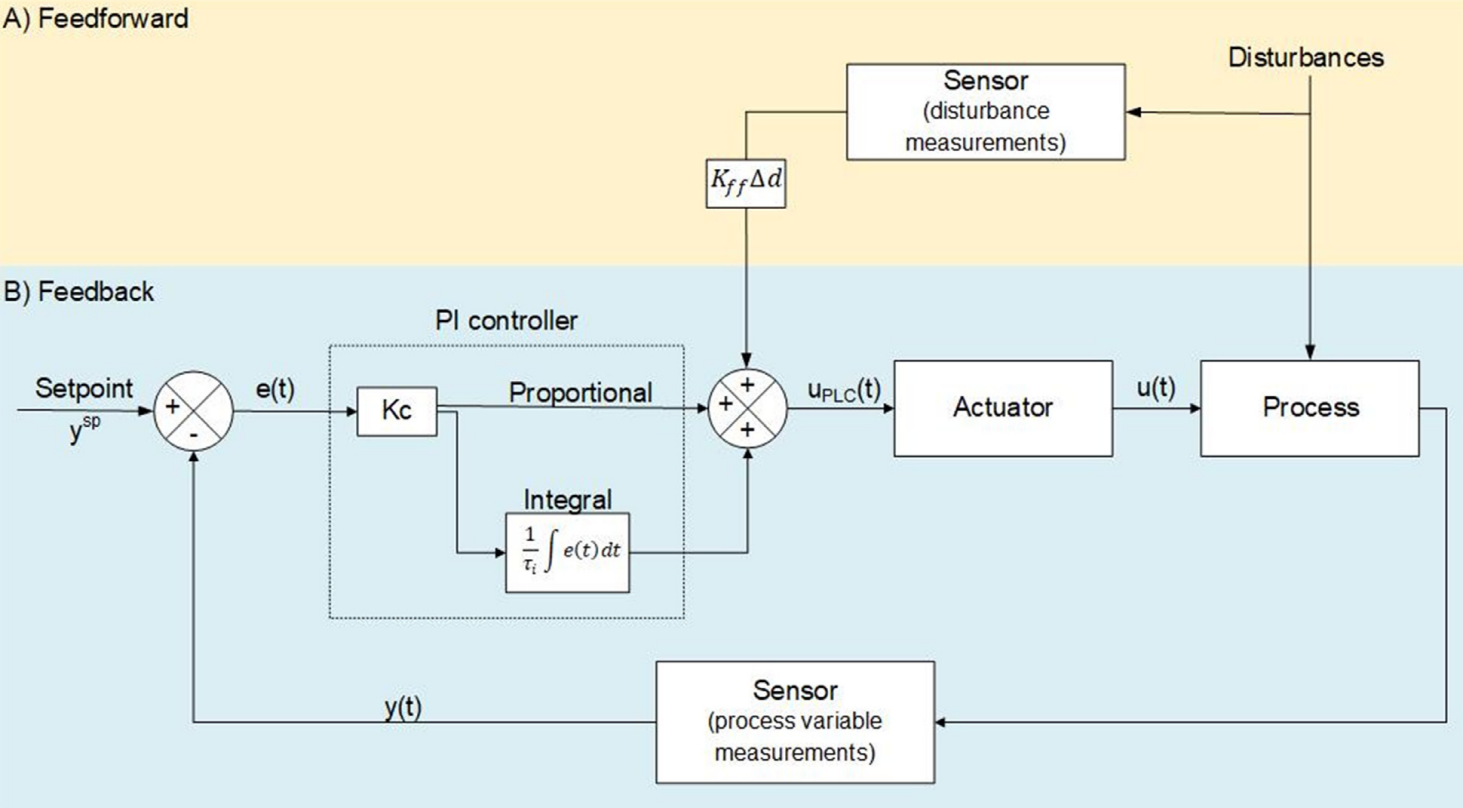
Schematic of a feedback-feedforward control strategy. Schematic A) shows the feedforward control strategy, where $K_{\text{ff}}$ is a proportional constant Δd is the difference in disturbances (see Eqs. (7) and 8). In this study, the disturbances are the inlet flowrates in each flotation cell. Schematic B) shows the feedback control strategy. In this study, $y^{\text{sp}}$ refers to level setpoint, y(t) is the level measurements, $u_{\text{PLC}}\left(t\right)$ is the signal sent to the PLC in bits to change the rpms in the peristaltic pump, and u(t) is the actual tailings flowrate.

Feedback control is a widely used strategy that can be found in most industries. This strategy usually uses a PID control algorithm (Eq. (1)) to calculate the corresponding output value. The calculated output is then sent as a signal to the process. A feedforward control strategy is used to reject persistent disturbances that cannot be rejected with feedback control. The feedforward control strategy is rarely implemented alone; instead, it is added to the feedback control, as depicted in Fig. 13.

The performance of a control system can be improved significantly by the application of feedforward control if the disturbance greatly affects the controlled variable, and if the disturbance can be measured. Feedforward controllers then require an online measurement of the disturbance to effectively reduce the effect on the controlled variable.

In this study, the inlet flowrate of each cell was considered a measured disturbance. This means that the feed flowrate was considered as the disturbance for cell 1, while the tailings flowrate from cell 1 was considered as the disturbance for cell 2, and the tailings flowrate of cell 2 was considered the disturbance of cell 3. To include the disturbance measurements in the feedback controller, Eq. (1) was modified as follows:

(7) $$u_{i}\left(t\right)=K_{c_{i}}\left(e_{i}\left(t\right)+\frac{1}{\tau_{I_{i}}}\int_{0}^{t}e_{i}\left(\tau \right)d\tau \right)+K_{ff_{i}}\Delta Q_{i}\;\;\;\;\;\;\mathrm{for}\;\;\;\;\;i=1,2,3.$$

As can be seen in Eq. (7), only a Proportional-Integral (PI) controller was used for each cell i, for i=1,2,3(i.e. $\tau_{d}=0$ in Eq. (1)). The additional term $K_{ff_{i}}\Delta Q$ corresponds to the feedforward strategy, where:

(8) $$\Delta Q_{d_{i}}=Q_{\mathrm{di}}\left(t\right)-Q_{d_{i}}\left(t-1\right)\;\;\;\mathrm{for}\;\;\;i=1,2,3,$$

where $Q_{d_{i}}$ is the flowrate disturbance of each cell. The constants $K_{ffi}$ were considered as proportional constants between 0 and 1 for each cell. The final value of these constants for each cell was obtained by trial and error, from which it was found that the best constant values were: $K_{ff}{{}_{cell}}_{1}=K_{ff}{{}_{cell}}_{2}=0.8,$and $K_{ff}{{}_{cell}}_{3}=1$.

## Method validation: results of the control implementation in the experimental system

The control experiments were codified in MATLAB, using the corresponding functions to read/write from/to the PLC (see Fig. 7). The air flowrate was set at 47 lpm (i.e., superficial gas velocity $j_{g}$ = 0.87 cm/s for these cells) in each cell for 5 min to ensure steady state. Setpoint arrays were created for the level of each cell. A *while* loop was used to perform the control strategy for a given set of tuning parameters. Each experiment was performed for 15 min with a sampling time of 1 second. Within the *while* loop, the optical level sensor signals were read using *ddereq*, which were filtered using a median moving filter, using *medfilt1* with a window size of 10 s. With this information, the control signals were calculated using Eq. (1), and were sent to the peristaltic pumps using *ddereq*.

Fig. 13 depicts the level control for each flotation cell using a feedback-only control strategy (left graphs) and a feedback-feedforward control strategy (right graphs). The control implementation was performed using the tuning parameters shown in Table 1. The Integral Absolute Error (IAE) was calculated using Eq. (9) for each set of experiments to compare them not only qualitatively, but also quantitatively.

(9) $$\text{IA}E_{i}=\int_{0}^{t}\left|e_{i}\right|\mathrm{dt}\;\;\;\mathrm{for}\;\;\;i=1,2,3.$$

**Fig. 14:**
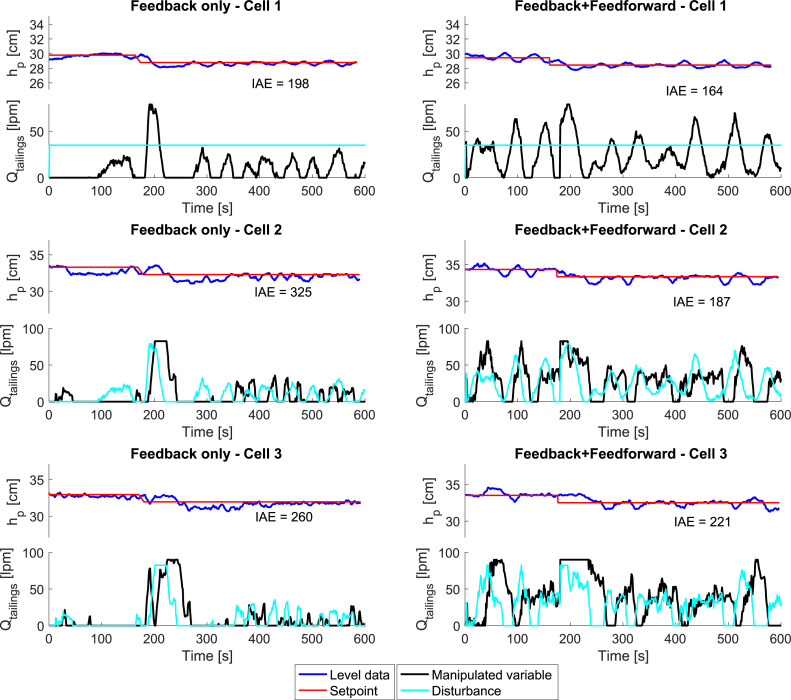
Level control for each flotation cell. The graphs on the left correspond to the implementation of a feedback-only control strategy, while the graphs on the right correspond to the implementation of a feedback-feedforward control strategy. The red lines are the level setpoints, and the blue lines are the filtered level data, which were read from the sensors in real-time. The manipulated variables of each flotation cell, represented as black lines, are the peristaltic pumps that modify the tailings flowrates. The disturbances are represented as cyan lines. The Integral Absolute Error (IAE) for each experiment is displayed.

**Fig. 15:**
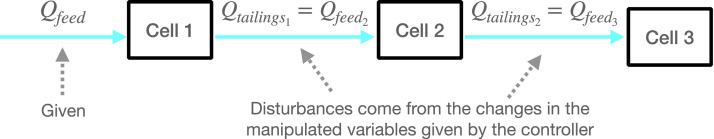
Schematic of the disturbances in the experiments.

The results of the control implementation using feedback-only, and feedback-feedforward strategies are depicted in Fig. 14. The disturbances applied to these experiments correspond to the feed flowrate ($Q_{feed}$) in each cell (cyan lines in Fig. 14). For cell 1, the feed flow was maintained constant at 45 lpm, while in cells 2 and 3, the disturbances came from the tailings flowrates of cells 1 and 2, respectively, as illustrated in Fig. 15.

The results depicted in Fig. 14 shows that the IAE value is lower in all cases when using a feedback-feedforward control strategy, which improved from 33 to 138 cm s. These improvements reflect the potential of the feedforward strategy for processes in which the disturbances can be measured, as in this case. It must be noted, though, that the disturbances in cells 2 and 3 had more fluctuating disturbances as the feed flowrates for those cells were the tailings flowrate from the previous cell (see Fig. 15). These differences imply that the controller in cells 2 and 3 must be sufficiently well tunned to allow for disturbance rejection.

A comparison of the feedback-feedforward strategy with the feedback-only strategy (Fig. 14) reveals greater variability in the manipulated variables. These variations can be caused by the feedforward control strategy compensating for disturbances so the pulp level setpoint can be reached more quickly. Furthermore, it must be noted that both the lower and upper limits of the manipulated variables were constrained in accordance with the ranges permitted by the peristaltic pumps. The rate of change of the manipulated variables was not constrained because the peristaltic pumps were able to adapt to abrupt changes.

## Conclusions

A methodology for a laboratory-scale level control strategy for an integrating system was described. The experimental system consisted of a bank of three cells connected in series, which mimics the froth flotation systems found in mineral processing plants. The experimental system was defined as an integrating system because the level control was performed using peristaltic pumps, instead of the classic control valves. Unlike the self-regulating dynamics of the traditional control valve systems, the use of peristaltic pumps makes it more challenging to calibrate the tuning parameters of the controller. Therefore, a detailed methodology to design a Proportional-Integral-Derivative controller was described, by following the “Simple Internal Model Control” (SIMC) tuning rules. The tuning parameters were then validated with experimental data using a classical feedback control strategy, as well as a feedback-feedforward control strategy, in an online fashion. The results showed that the level control performance significantly improves when a feedforward strategy is considered because it allowed a better account for the process disturbances. The advantage of using this method lies in its potential application to validate and further improve control strategies for the froth flotation process.

## Additional information

The process can be approximated as a first-order linear differential equation:

(10) $$\tau_{1}\frac{dy\left(t\right)}{dt}=-y\left(t\right)+ku\left(t-\theta \right),$$

where y(t) corresponds the output (pulp level) and u(t−θ) is the input (tailings flowrate) with time delay (θ) in each flotation cell. Taking the Laplace transform to each of the terms in Eq. (10), we obtain:

(11) $$L\left(\tau_{1}\frac{dy\left(t\right)}{dt}\right)=L\left(-y\left(t\right)\right)+L(ku\left(t-\theta )\right),$$

where:

(12) $$L\left(\tau_{1}\frac{dy\left(t\right)}{dt}\right)=\tau_{1}sY\left(s\right),$$

(13) L(−y(t))=−Y(s),

(14) $$L(ku\left(t-\theta )\right)=kU\left(s\right)e^{-\theta s}.$$

Replacing Eqs. (12) to 14 into Eq. (10) yields:

(15) $$\tau_{1}sY\left(s\right)=-Y\left(s\right)+kU\left(s\right)e^{-\theta s}.$$

Reordering the terms in Eq. (15) to obtain the transfer function in its conventional form $G\left(s\right)=\frac{Y(s)}{U(s)}$ we obtain Eq. (4), i.e.:

$$G\left(s\right)=\frac{Y\left(s\right)}{U\left(s\right)}=\frac{k}{\tau_{1}s+1}e^{-\theta s}.$$

## CRediT authorship contribution statement

**Paulina Quintanilla:** Conceptualization, Methodology, Investigation, Funding acquisition, Software, Validation, Data curation, Visualization, Writing – original draft. **Daniel Navia:** Conceptualization, Methodology, Investigation, Software, Validation, Writing – review & editing. **Felipe Moreno:** Investigation, Writing – review & editing. **Stephen J. Neethling:** Supervision, Formal analysis, Writing – review & editing. **Pablo R. Brito-Parada:** Supervision, Formal analysis, Funding acquisition, Writing – review & editing.

## Declaration of Competing Interest

The authors declare that they have no known competing financial interests or personal relationships that could have appeared to influence the work reported in this paper.

## Data Availability

Data will be made available on request.
